# Fatigue in an AZ31 Alloy Subjected to Rotary Swaging

**DOI:** 10.3390/ma15217541

**Published:** 2022-10-27

**Authors:** Zuzanka Trojanová, Zdeněk Drozd, Kristýna Halmešová, Ján Džugan, Tereza Hofrichterová, Peter Palček, Peter Minárik, Tomáš Škraban, František Nový

**Affiliations:** 1 Faculty of Mathematics and Physics, Charles University, Ke Karlovu 3, 121 16 Praha, Czech Republic; 2 Comtes FHT, Průmyslová 996, 334 41 Dobřany, Czech Republic; 3 Department of Materials and Engineering, University of Žilina, Univerzitná 1, 010 26 Žilina, Slovakia; 4 Research Centre, University of Žilina, Univerzitná 8215/1, 010 26 Žilina, Slovakia

**Keywords:** magnesium alloy AZ31, rotary swaging, fatigue life, twinning, non-basal slip, fractography

## Abstract

The magnesium AZ31 alloy was swaged with rotary pressure with the aim of redefining the microstructure and improving mechanical and fatigue properties. The rotary swaging process and subsequent ageing improved the yield stress in tension and compression. In the present study, the investigation was focused on fatigue behaviour. The samples were cycled in a symmetric regime with a frequency of 35 Hz. A dependence of the stress amplitude on the number of cycles up to the fracture was estimated. The microstructure of the samples and fracture surfaces was analysed with a scanning electron microscope. The fatigue process was influenced by the pronounced texture formed in the swaging process. The fatigue properties of the swaged samples improved substantially—the endurance limit based on 10^7^ cycles was approximately 120 MPa—compared to those of the cast alloy. The analysis of the fracture surfaces showed a transcrystalline fatigue fracture.

## 1. Introduction

In recent decades, new processing methods have been developed to improve the mechanical properties of magnesium alloys. Severe plastic deformation (SPD) methods, such as equal channel angular pressing (ECAP), accumulative roll bonding (ARB), rotary swaging (RSW), high pressure torsion (HPT), and possibly methods derived from them or a combination thereof [1]. These methods can substantially reduce grain size and effectively contribute to material strengthening [2,3,4]. Magnesium alloys manufactured by SPD methods generally exhibit a certain texture, tension compression asymmetry, and pronounced anisotropy of physical properties [4,5,6,7,8]. This behaviour is a consequence of the hexagonal closed-packed magnesium (hcp) structure with a lack of crystallographic equivalent. The five independent slip systems, necessary for compatible deformation [9], can be made up of basal <a> dislocations and non-basal <a> and <c+a> dislocations movable in the prismatic and pyramidal planes. Note that the basal texture may be wakened by a choice of suitable alloying elements, usually rare earth elements and the SPD technique [10]. The mechanical and fatigue properties of AZ magnesium alloys have been reported in the literature in many studies. The strength of the alloys increases with increasing Al content, whereas ductility decreases. The magnesium alloy AZ31 with a low content of Al can be processed with various types of SPD processes with the aim of manufacturing materials with improved (and in many cases predetermined) mechanical properties. The fatigue properties of AZ magnesium alloys, manufactured in various techniques, were cited in several articles [11,12,13,14,15,16,17,18,19,20]. The authors demonstrated that metallurgical factors and microstructure are the deciding factors for the fatigue life of alloys, especially in the low-stress amplitude region. Matsuzuki and Horibe [21] studied deformation processes in the cycled magnesium alloy AZ31. They estimated that dislocation slip is the dominant deformation process at lower strain amplitudes, while mechanical twinning is the predominant deformation mode at higher strain amplitudes. Horynová et al. [22] studied fatigue behaviour of the squeeze cast AZ31 alloy under the stress amplitude control mode. The authors of [21] did not observe any tension–compression asymmetry of the hysteresis loops. The crack initiation started from the sample surface or from clusters of secondary phase particles. The final fracture occurred in the transgranular mode. The fatigue properties of the AZ91 alloy processed by ECAP were studied by Fintová et al. [23]. They observed an improvement in mechanical properties in tension and compression compared to those of cast alloy, and an improvement in fatigue life in the low cycle region, while the improvement in fatigue life in the high cycle region was marginal. Wrought magnesium alloys usually exhibit a basal texture, where the basal planes are parallel to the extrusion or rolling directions. The consequence of this texture is a pronounced tension–compression asymmetry. YJ Kim et al. [24] investigated the fatigue properties of AZ31 sheets of different thickness manufactured with twin roll casting and subsequent hot rolling. The authors found an asymmetric hysteresis loop at strain amplitudes greater than 0.6%. This finding is due to the formation of extension twins $\left\{10\bar{1}2\right\}\;\langle 10\bar{1}1\rangle$ in the compressive half cycle, while detwinning and subsequent slip occurred in the tension half cycle over the whole fatigue life. Wang et al. [25] reported that the AZ31 rolled magnesium alloy exhibits the highest and lowest fatigue strength when cyclic loading is applied along the rolling direction and 45 ° away from the rolling direction. The randomly oriented and textured alloy of AZ31 was strained by various types of cyclic stress [26]. Similarly, twinning occurred in both randomly oriented samples and extruded samples with pronounced basal texture under compressive stress. Detwinning was observed in textured samples under a tensile stress that was lower than the yield stress in the one-way test. To elucidate the role of twinning in the plane of the initiation of fatigue crack, bending fatigue tests were conducted in various stress ratios [27]. The authors of [26] estimated that twinning is not involved in the initiation of cracks in all cases. Large grains with a high Schmid factor oriented to basal slip may be sites of crack initiation. Jamali et al. [28] investigated the influence of grain size and grain boundary misorientation on crack initiation in a textured alloy AZ31. They found that cracking of large grains and grain boundaries with a large angle of misorientation is the dominant fracture mechanism. Ayer studied the influence of the extrusion ratio on the microstructure and mechanical and fracture properties of the AZ31 alloy [29]. They estimated that the increased extrusion ratio refines the microstructure and improved the strength. The fracture surfaces exhibited a ductile and brittle mixture. The fracture mechanism was not influenced by extrusion parameters [29]. Hot-rolled AZ31 sheets were subjected to dynamic plastic deformation [30]. This type of deformation produced many twins that formed lamellae. This twin lamellar structure fractured in the dimple mode, while samples without dynamic deformation exhibited a cleavage fracture with a river pattern. 

Various processes of severe plastic deformation have improved the mechanical and fatigue properties of the AZ31 alloys [23,24,25]. In our previous article [30], we studied the tensile and compressive properties of an AZ31 alloy subjected to the rotary swaging process. The tensile properties of the swaged materials were substantially improved. The goal of the present work is to reveal the relationship between microstructure and fatigue behaviour of a rotary-swaged magnesium alloy via a combination of cycling experiments and EBSD observations. Consequently, an analysis of the fracture surfaces is also performed.

## 2. Materials and Methods

Commercially available extruded rods from AZ31 magnesium alloy (nominal composition: 3 wt% Al, 1 wt% Zn, 0.3 wt%Mn, balance Mg) were used for the rotary swaging (RSW) process. The as-received material was heated up to 400 °C and stepwise rotary swaged five times from the initial diameter of 20 mm down to 8 mm in a rotary swaging machine HMP4-4 (Felss, Bretten-Gölshausen, Germany).

**Figure 1:**
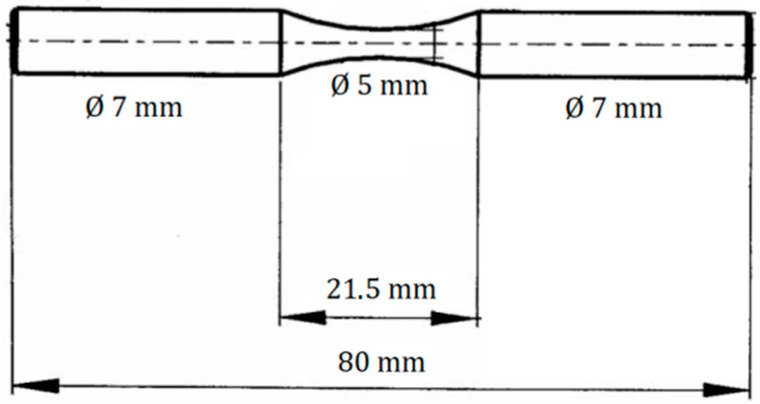
The shape and size of the samples for fatigue experiments.

Afterwards, the swaged rods were naturally aged at room temperature for 350 days. The grain structure of the extruded and swaged cylindrical samples was investigated by light microscopy (Zeiss Axio Observer 7 microscope, Carl Zeiss QEC GmbH, Munich, Germany) performed with an Olympus microscope (Olympus, Tokyo, Japan). In addition, electron backscatter diffraction (EBSD) analysis was used to analyse grain structure, texture, and local misorientation. The Zeiss Auriga Compact scanning electron microscope (Carl Zeiss microscopy GmbH. Oberkochen, Germany) equipped with the EDAX EBSD camera (EDAX, Pleasanton, CA, USA) and OIM 7.3 software was used for this analysis. Texture analysis was performed using the series expansion of generalised spherical harmonics under the axial symmetry condition. The kernel average misorientation (KAM) was used in the EBSD analysis as a measure of local grain misorientation. Fracture surfaces after cyclic loading were analysed with the use of Vega LMU II SEM (TESCAN ORSAY HOLDING, Brno, Czech Republic). Residual elastic strains were measured in an argon atmosphere using the Netzsch 410 dilatometer (Netzsch-Gerätebau GmbH, Selb, Germany), over a temperature range from room temperature to 400 °C. Aged RSWed samples were used for deformation and fatigue experiments. Tensile and compression tests were performed at room temperature on an Instron 5882 testing machine (Instron ltd, High Wycombe, UK), at an initial strain rate of the order of 10^−3^ s^−1^. The samples for tensile tests had a dog bone shape with a gauge length of 25 mm and a diameter of 6 mm. The samples for compression tests had a cylinder shape with a diameter of 6 mm and a height of 10 mm. Characteristic stresses, such as tensile/compression yield stress (TYS/CYS) and maximum tensile strength (UTS) and peak compression strength (PCS), were estimated as a true stress at the strain ε = 0.002 and the maximum true tensile/compressive stress. The standard deviation of the measured stresses was ±5%. Fatigue tests were performed on the Instron M22-1528 ElectroPuls E100003 fatigue machine (Instron, Norwood, MA, USA) at room temperature at a frequency of 35 Hz. Symmetrical cyclic push–pull loading (parameter of cycle asymmetry parameter (R = −1) at ambient temperature was used with a predetermined stress amplitude. The number of cycles was fixed at 10^7^ cycles. The stress amplitude was reduced stepwise from 150 MPa to 120 MPa. For one amplitude, two samples were tested. The geometry of the samples for the fatigue tests is shown in Figure 1. The surface of the samples was polished before cycling. Microhardness was measured with the Vickers indenter at room temperature in a Qness 10a apparatus (ATM Qness GmbH, Golling, Austria), with a load of 0.1 kg and a dwell time of 10 s. The microhardness was estimated from the section perpendicular to the extrusion direction.

## 3. Results

### 3.1. Microstructure of Samples

**Figure 2:**
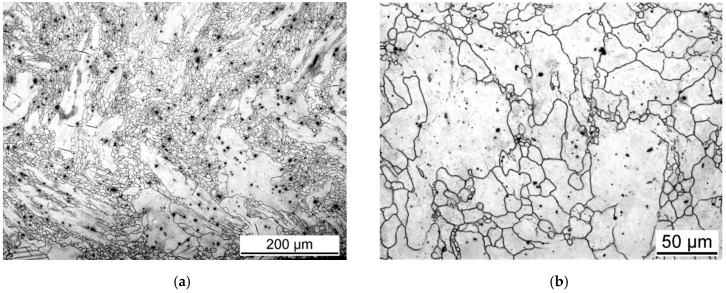
Microstructure of the initial (extruded) condition taken from the section perpendicular to the extrusion direction (**a**), with higher magnification of the same place (**b**).

**Figure 3:**
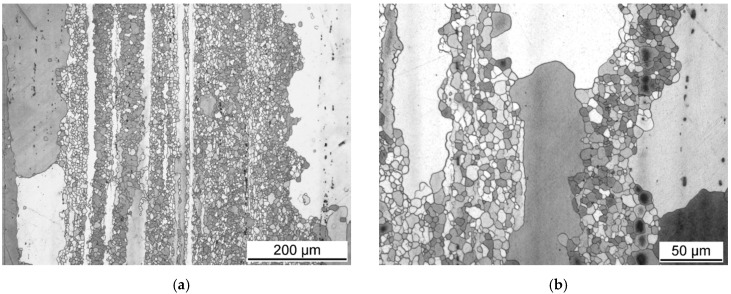
Microstructure of the initial (extruded) condition taken from the section parallel to the extrusion direction (**a**), with higher magnification of the same place (**b**).

The microstructure of the hot extruded AZ31 magnesium alloy taken from the section perpendicular to the extrusion direction is reported in Figure 2. The grain structure is nonuniform with the occurrence of large grains (~300 µm) surrounded by belts of small ones (~20 µm). In the section parallel to the extrusion direction (Figure 3), it is clear that the large grains are elongated in the extrusion direction, and the small grains are equiaxed. Such a microstructure is typical for the partial dynamic recrystallisation (DXR) that often occurs during the hot extrusion of magnesium alloys. Secondary phase particles, the black dots in Figure 2 and Figure 3, are aligned in the extrusion direction, indicating that they were present already in the material prior to the extrusion. 

**Figure 4:**
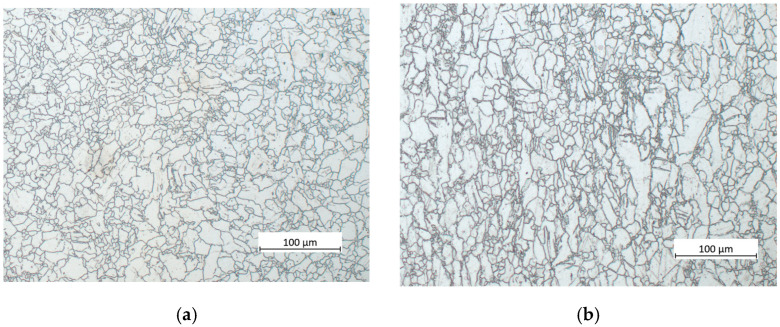
Light micrographs of the sample after quintuple swaging taken from the perpendicular (**a**) and parallel (**b**) to the rotary swaging direction.

Light micrographs of the processed material, after quintuple swaging steps, are shown in Figure 4a from the section perpendicular to the extrusion direction, and in Figure 4b from the section parallel to the extrusion direction. It is obvious that the grain structure became homogeneous and the grain size became smaller. The average grain size of the swaged sample was calculated in the previous study to be ~16 μm [31,32]. Note that the presence of large non-recrystallised grains observed in the as-extruded state was completely suppressed by the rotary swaging process.

**Figure 5:**
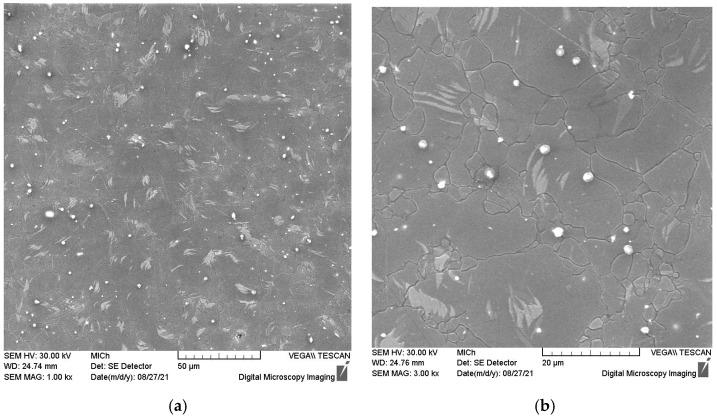
Scanning electron micrograph taken from the perpendicular section of the swaged sample (**a**) and from the higher magnification (**b**).

**Figure 6:**
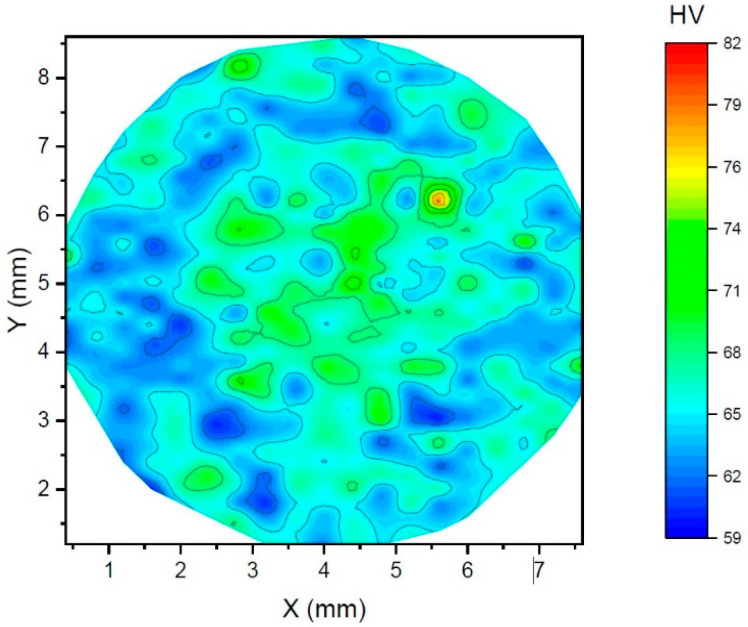
Microhardness map taken from the perpendicular section of the sample.

Scanning electron microscopy was used for identification of the secondary phase particles observed under all conditions of the studied material. Figure 5 shows a SEM micrograph with bright particles. The chemical analysis of the particles showed that they contain Mn; therefore, the particles are most likely Al8Mn5 precipitates, which are commonly observed in alloys of AZ series, containing a small addition of Mn for grain refinement [33]. Note that γ-Al12Mg17 intermetallic compounds, typical for magnesium alloys of AZ series [34], were observed neither in a light microscope nor in a scanning electron microscope. The uniformity of the microstructure refinement due to the swaging process was investigated via microhardness measurement. The microhardness map of the swaged sample is shown in Figure 6. It is obvious that the hardness is nearly uniform within the sample diameter. This result is different from the findings of other authors, who found significantly finer grains in the peripheral parts of the swaged rods, while in the central parts, much higher grains were found [35].

**Figure 7:**
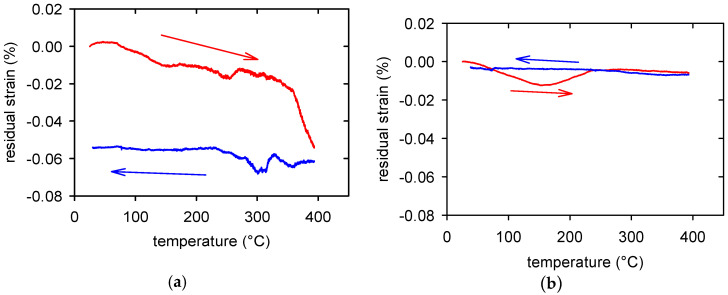
Temperature dependence of the residual strain while heating and cooling for swaged (**a**) and swaged and aged material (**b**).

In our previous studies, the same material as the one presented here was used only without the ageing procedure [31,32]. The swaged material exhibited significant internal tensile stresses in the extrusion direction. This is obvious from Figure 7a, where the residual strain is reported as a function of the temperature during heating and cooling up to 400 °C. However, the aged material exhibited only a minimum residual strain, as shown in Figure 7b. 

**Figure 8:**
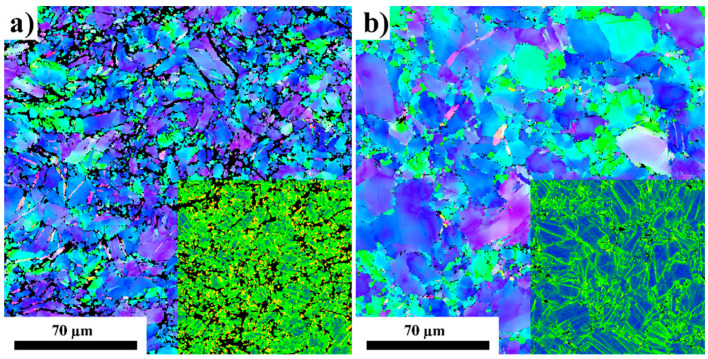
EBSD orientation maps of swaged (**a**) and swaged + aged (**b**) samples. Overlay of kernel average misorientation maps is presented in the bottom right corner of each orientation map (colour scale blue-green-red).

The microstructural difference between the swaged and subsequently aged samples was further investigated by EBSD. Figure 8 shows the EBSD orientation maps measured on the section perpendicular to the extrusion direction with overlay of the kernel average misorientation (KAM) maps. It is clear that the as-swaged sample exhibited relatively high residual strain, manifesting itself as a high number fraction of green-to-red pixels in the corresponding KAM map. On the other hand, significantly lower residual strain was observed in the aged sample. This result agrees well with the residual strain measurement shown in Figure 7. Note that the EBSD map of the swaged sample contained a relatively high number of black pixels in which the indexation failed due to too high local strain. The EBSD analysis also showed that the average grain size did not significantly change because of the ageing and was ~16 μm in both samples. 

**Figure 9:**
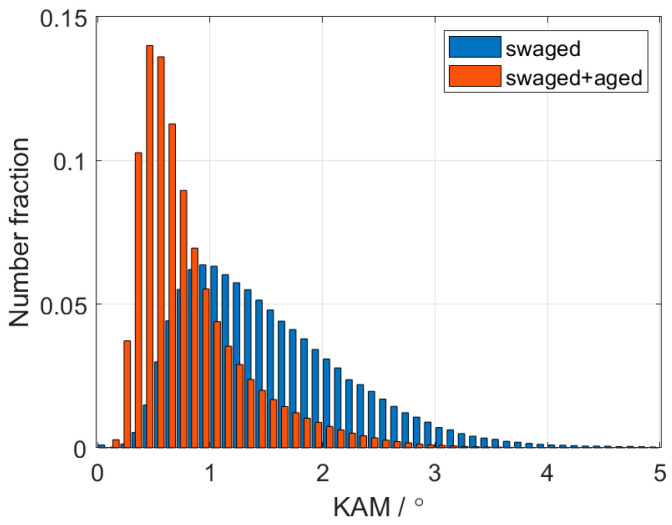
KAM distributions of the swaged and swaged + aged samples.

Figure 9 presents the KAM distributions for the swaged and aged samples calculated from the EBSD data. The analysis showed a distinct shift of the KAM parameter to lower angles after ageing, and the whole distribution became narrower. This experimental fact indicates massive recovery of the dislocation substructure, where the dislocations found their places in the dislocation walls with a lower position energy. Recovery of the dislocation substructure is also evident in Figure 8b compared to Figure 8a.

**Figure 10:**
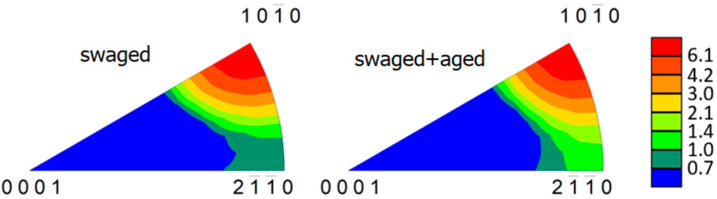
IPFs of the swaged and aged samples calculated from the EBSD data. The extrusion direction is perpendicular to the plane.

The inverse pole figure (IPF), calculated for the section perpendicular to the extrusion direction, is shown for the swaged and aged samples in Figure 10. The swaged sample exhibits a strong $\left\{10\bar{1}0\right\}$ fibre texture, which is typical for the extruded and swaged magnesium alloys due to axially symmetric radial compression deformation. The data unambiguously showed that the ageing process did not cause any major change in the crystallographic texture of the investigated material. 

### 3.2. Deformation Curves

**Figure 11:**
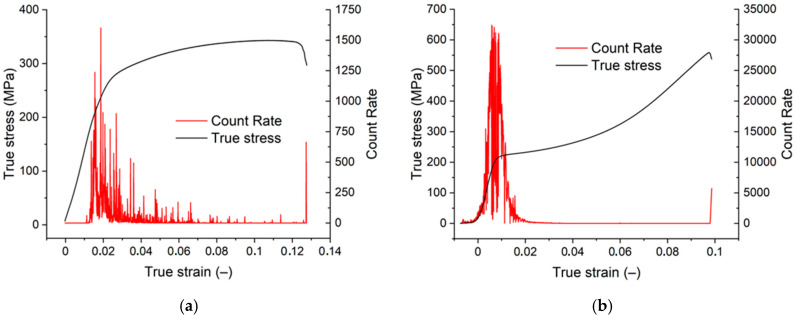
True stress–true strain curves obtained in tension (**a**) and compression (**b**).

Tensile and compression deformation curves are shown in Figure 11a,b. The characteristic stresses and the total strain are reported in Table 1. For comparison, the values found for the original extruded material are given in Table 1. During deformation, acoustic emission was recorded. It is obvious that the acoustic emission signal is much stronger in compression. 

The estimated values showed that the swaging process and subsequent ageing substantially improved both the tensile and compressive deformation stresses. 

### 3.3. Fatigue

**Figure 12:**
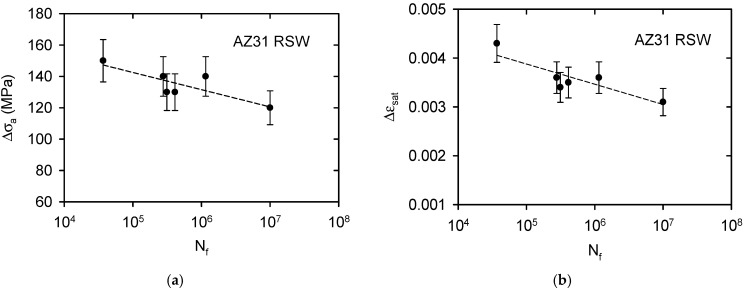
Relation between the stress amplitude and the number of cycles to fracture (**a**), the strain amplitude, and the number of cycles to fracture (**b**).

As mentioned above, the samples were cyclically loaded in the push–pull regime under a sinusoidal stress cycle. The stress amplitude was reduced stepwise from 150 MPa to 120 MPa. Samples loaded with an amplitude of 120 MPa were not broken even at the limit number of cycles 10^7^. This amplitude may be considered as the fatigue limit. Figure 12a shows the variation of the stress amplitude Δs, as well as the number of cycles to failure, N_f_, on the semi-logarithmic scale. Similarly, the dependence of the saturated strain amplitude on the number of cycles, N_f_, is shown in Figure 12b.

### 3.4. Fracture Surface

**Figure 13:**
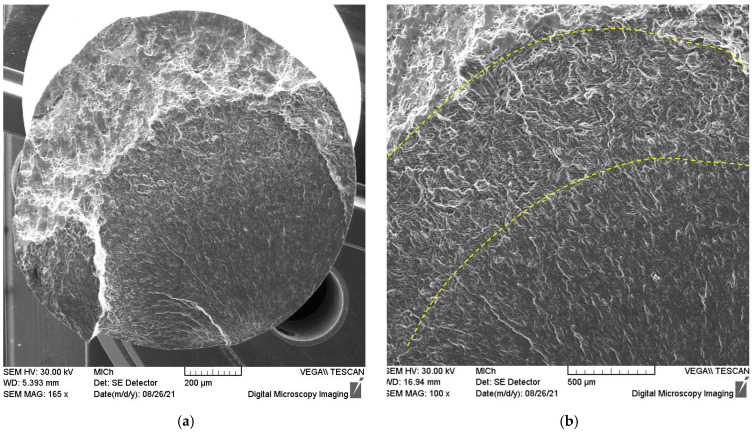
Overall SEM view of the fracture surface (**a**): the transition region on the fracture surface is delineated by the yellow lines (**b**).

**Figure 14:**
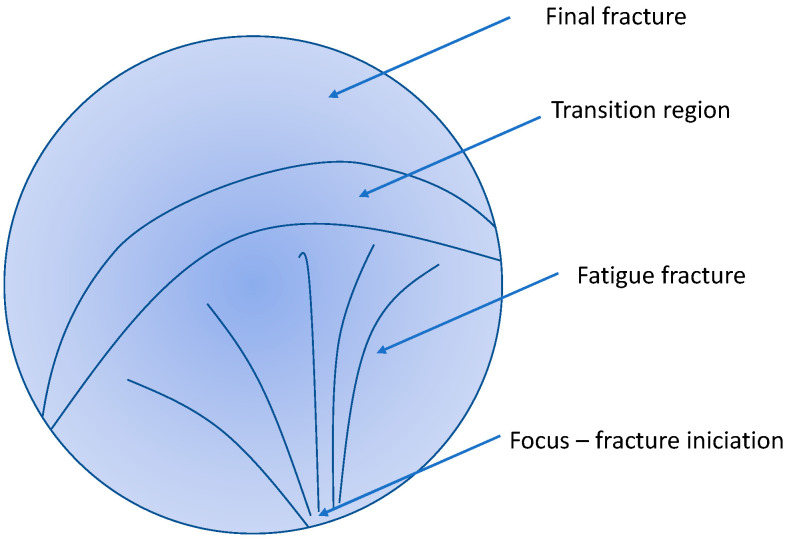
Schematic drawing of the fracture surface showing the observed zones.

The general view of the fracture surface of the broken sample after the fatigue test is shown in Figure 13a. It is obvious that the fracture surface exhibits three zones, as illustrated in Figure 14. 

The part of the surface created due to the growth of stable fatigue cracks represents approximately 50% of the entire fracture area. This region is followed by the transition region with a rapid unstable growth of the magistral fatigue crack, where it is possible to find a mixture of fatigue and ductile failure (see Figure 13b). The final fracture with the characteristic ductile fracture can be seen in the upper left corner of the image shown in Figure 13a.

**Figure 15:**
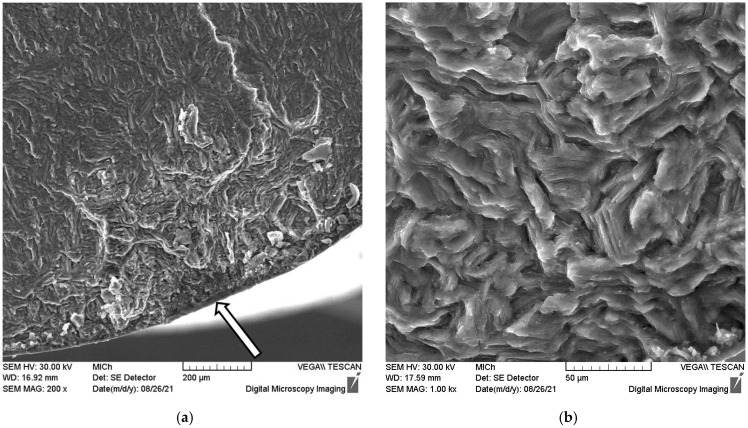
The initiation point of the fatigue crack (**a**) and the detail of the fatigue fracture surface close to the initiation point (**b**).

**Figure 16:**
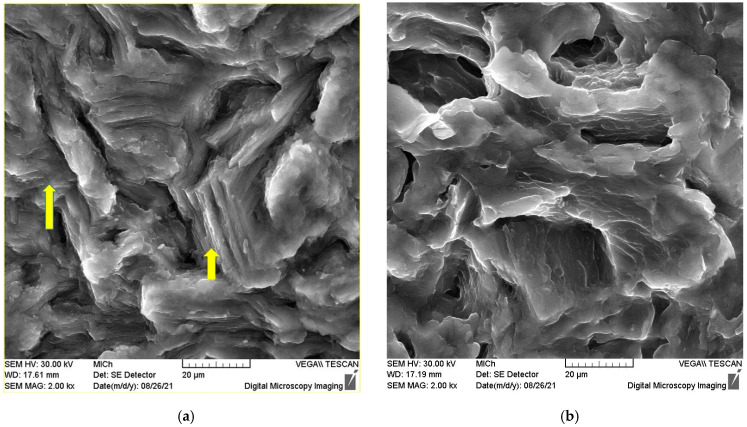
Deformed grains on the surface of the fracture and traces of the basal planes (**a**), river morphology, and secondary cracks on the surface of the fracture (**b**).

The initiation point of the fatigue crack is visible in the lower part of the fracture surface in the vicinity of the sample surface. The surface of the fatigue fracture, with the origin of the fracture in the bottom part of the micrograph, is shown in Figure 13a,b. It is obvious that the origin of the fracture is some surface imperfection. The transgranular fatigue fracture near the initiation point is characterised by many star-shaped striations. The fatigue crack does not propagate continuously from the focus. An additional deformation of the free fracture surface released internal stress, which is visible in Figure 15b and Figure 16a. 

Slip traces in the deformed grain show exposed basal planes indicated by arrows in Figure 16a. From the picture, it is obvious that the basal planes are oriented perpendicular to the fracture surface. Small twins can be observed in the upper corner of the picture. The quasi-cleavage facets and river patterns are visible in Figure 16b together with deep secondary cracks. 

**Figure 17:**
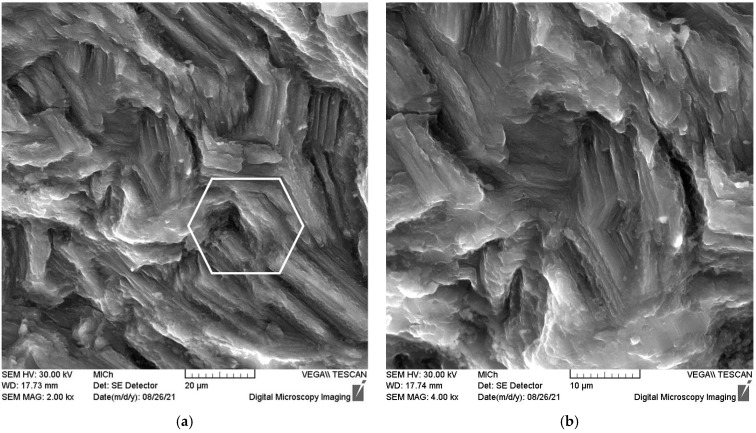
Fatigue fracture surface with uncovered basal planes (**a**) and fatigue striations (**b**).

The inscribed hexagon in Figure 17a shows that the angle between the basal planes is 120°. Many fatigue striations are visible in Figure 17b.

**Figure 18:**
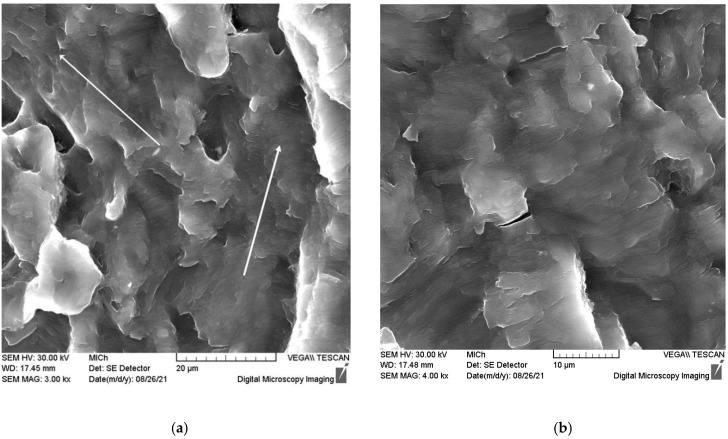
Scanning electron micrograph of the fracture surface taken from the transition region (**a**) at the same place with a higher magnification (**b**).

The fracture surface taken from the transition region is shown in Figure 18a,b. From Figure 18a, it is obvious that the fracture propagated in two directions indicated by white arrows. Using a higher magnification, fine fatigue striations are visible in Figure 18b. It is obvious that the number of secondary cracks increased in the transition region. 

**Figure 19:**
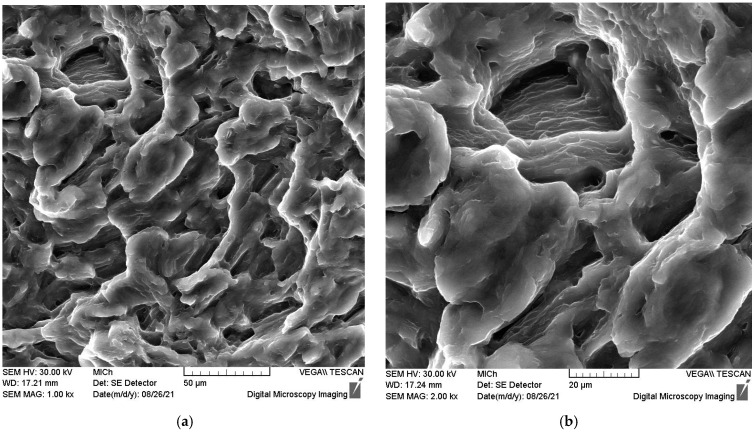
Scanning electron micrograph taken from the transition region (**a**), dimple on the fracture surface with fine striations inside (**b**).

Equiaxed dimples, which can be expected in tension, are visible in the transition region in Figure 19a. The high-magnification view in Figure 19b shows the detail of a dimple with fine striations inside.

## 4. Discussion

### 4.1. Fatigue Mechanisms

The original inhomogeneous microstructure of the AZ31 alloy was completely rebuilt in the RSW process. The resulting material exhibits fine equiaxed grains. The texture in which the basal planes (0001) are parallel to the swaging direction is more pronounced compared to the texture of the original bars. 

The quasistatic stress–strain curves obtained in tension and compression presented in Figure 11a,b show a substantial improvement in TYS and CYS compared to the extruded material. Although the ultimate tensile stresses of all samples (extruded, swaged, and swaged+aged) are comparable, the compression peak stresses significantly increased in swaged and swaged+aged samples. The increase in TYS and CYS may have mainly two reasons: (a) decrease in grain size in the swaged samples and (b) texture of samples. The isothermal upsetting further improved mechanical properties, as is shown in Table 1.

The influence of grain size on deformation stresses is expressed in the Hall–Petch model, where grain boundaries are considered impenetrable obstacles to dislocation motion [36]:

(1) $$\sigma_{\varepsilon }=\sigma_{0\varepsilon \;}+k_{\varepsilon }\;d^{-1/2}$$

where σ_ε_ is the true stress at the true strain ε, σ_0ε_ is the friction stress, k_ε_ is the intensity of microstructural stress, and d is the average grain diameter. A similar philosophy can be used for twin boundaries as obstacles to dislocation motion [37,38].

(2) $$\sigma_{t}=\sigma_{0t}+k_{t}d^{-1/2}$$

where the indices t’s are related to twinning. 

Maximum AE signal strength was observed in both tension and compression in the vicinity of yield stress (see Figure 11a,b). The main sources of AE are considered collective motion of dislocation ensembles and mechanical twinning [39,40]. The AE peak is much higher while deformed in compression. The stress–strain curve obtained under tension exhibits a typical parabolic shape. The basal texture that was formed during the rotary swaging process indicates that most of the basal planes are parallel to the extrusion direction. This orientation is not favourable for basal slip. The deformation process starts in the basal planes whose orientation allows for the slip of <a> dislocations and continues with the activity $\left\{10\bar{1}2\right\}$ $\langle \bar{1}011\rangle$ extension twins. The formation of twins reorients the basal planes by 86.3° and allows for the slip of dislocations in the basal planes [41]. The critical resolved shear stress for nucleation and formation of extension twins in randomly oriented polycrystalline Mg was found to be 15–40 MPa in tension and 10–30 MPa while deformed in compression [41]. Critical stress for contraction twins $\left\{10\bar{1}1\right\}$ $\langle 10\bar{1}2\rangle$ is higher than 76–153 MPa [42]. Reoriented basal planes still have a very low Schmid factor, but pyramidal planes of the first and second order are oriented much more favourably for the glide od <c+a> dislocations. The critical resolved shear stress (CRSS) for <a> dislocations in the $\left\{11\bar{2}1\right\}$ pyramidal planes were found in an AZ31 alloy sheets τ0 = 40 MPa while in the $\left\{11\bar{2}2\right\}$ planes of the second order τ0 = 45–81 MPa for <c+a> dislocations [43]. Cyclic deformation of the AZ31 sheet tension followed by compression showed that, under low stress, there was initial twinning and shrinkage of existing twins [44]. This mechanism does not require any accommodation by slip. The tensile behaviour of pre-deformed samples was studied by Bohlen et al. [45]. The authors estimated detwinning at the early stages of deformation followed by the generation of new twins. During cyclic deformation, this twinning–detwinning mechanism is probably exhausted. Lamark et al. [46] measured the acoustic emission (AE) produced during cyclic deformation of an AS21X alloy. The AE signal showed a strong asymmetry with respect to tension and compression deformation. The authors explained this result by massive twinning in the compressive half cycle. According to Agnew [47], twinning in the tensile half cycle can introduce significant compressive residual stresses in Mg that are retained after loading. This means that the Bauschinger effect is to be expected. Stress-controlled fatigue tests (R = −1) were performed at low frequencies (0.055–1 Hz) on an extruded alloy of AZ31 [48]. The authors of [48] found asymmetric hysteresis curves, whereas the asymmetry vanished in a stepwise fashion with increasing number of cycles. 

The endurance limit based on 10^7^ cycles for the AZ31 alloy was 120 MPa. This value is similar to the results of Zúberová et al. [20] estimated for the alloy AZ31 submitted to hot rolling. Note that the endurance limit found for the ECAPed AZ31 alloy was 80 MPa, and for squeeze cast AZ31 samples, it was only 40 MPa [20]. The strain amplitude increased with increasing stress amplitude and acquired a saturated value in all samples after approximately 150 cycles by ε = 0.003–0.004. Because the apparatus used did not allow for any measuring of the hysteresis loops, it was not possible to divide the strain amplitude into elastic and plastic components. 

The deformation mechanisms in the AZ31 alloy submitted to the rotary swaging process were studied using acoustic emission (AE) in [31]. Twinning was observed at the beginning of plastic deformation in both tension and compression tests. The twinning is followed by the dislocation motion in reoriented prismatic planes in tension, while in compression, the incoming mechanism is the growth of twins. In addition to the anelastic/plastic deformation mechanisms mentioned, sliding of the grain boundary (GBS) was observed as a further deformation mechanism in AZ31 rolled sheets [49]. The activation energy of this mechanism exhibits at room temperature 80 kJ/mol, that is, a value comparable to the diffusion of the grain boundary [49]. Slip–induced GBS may help to the compatible deformation when the plastic strain anisotropy is present by an interaction of lattice dislocations with grain boundaries. Its contribution to the total strain can be approximately 8%, that is, comparable to the contribution of twinning [50]. 

### 4.2. Fatigue Behaviour

If we fit the experimental points in Figure 10a by a straight line and apply the Manson–Coffin power law expression [51]

(3) $$\sigma_{a}=\sigma_{f}^{\prime }{\left(2N_{f}\right)}^{b}$$

We obtain the fatigue strength coefficient, $\sigma_{f}^{\prime }=\left(250\pm 30\right)\;\mathrm{MPa}$, and fatigue strength coefficient, b = (−0.036 ± 0.009). The relationship between strain amplitude and fatigue life, N_f_, may be expressed as

(4) $$\varepsilon_{a}=\varepsilon_{f}^{\prime }{\left(2N_{f}\right)}^{c}$$

where $\varepsilon_{f}^{\prime }$ is a fatigue ductility coefficient and c is the fatigue life exponent [52]. Using data reported in Figure 10b, the values are $\varepsilon_{f}^{\prime }$ = 0.0069 and for the ductility coefficient, c = −2.16 ± 0.059. The fatigue life can be predicted using the Smith–Watson–Topper (SWT) equation [53]

(5) $$\sigma_{a}\varepsilon_{a}=\frac{{\sigma_{f}^{\prime }}^{2}}{E}{\left(2N_{f}\right)}^{2b}+\sigma_{f}^{\prime }\varepsilon_{f}^{\prime }{\left(2N_{f}\right)}^{\left(b+c\right)}$$

where E is the Young modulus of the alloy. With knowledge of *σ*′_f_, *ε*′_f_, b, and *c*, the fatigue life can be predicted. Another approach based on energy dissipated during fatigue life was proposed in [54]. The strain energy per unit volume can be expressed as

Δ*E_t_* = *E*’*_e_*(2*N_f_*)*^h^* +*E*’*_f_* (2*N_f_*)*^g^* (6)

where *h* is the fatigue strength exponent, *g* the fatigue toughness exponent, and E′_e_ and E′_f_ are the fatigue strength coefficient and the fatigue toughness coefficient, respectively. The strain energy that is dissipated in one cycle can be expressed as the hysteresis loop area.

(7) $$\Delta W=\iint d\sigma_{a}d\varepsilon_{a}$$

where *σ*_a,_ and *ε*_a_ are the stress and strain amplitudes, respectively. This energy depends on the shape of the hysteresis loop; in the first approximation, this energy can be written as

(8) $$\Delta W=\kappa d\sigma_{a}d\varepsilon_{a}$$

where κ is a material constant that can be estimated experimentally. Then, the total mechanical energy that was during the fatigue life was mostly dissipated into heat.

(9) $$W=\Delta W=N_{f}\kappa d\sigma_{a}d\varepsilon_{a}$$

### 4.3. Fracture Mechanism(s)

Although the surface of the samples was specially prepared, the main fatigue cracks leading to the sample fracture started in all samples on the surface of the sample (see Figure 15). The fracture area morphology consists of four zones (focus, stable crack growth, transition region of unstable crack growth, and final rupture). The stable propagation zone of the fatigue crack starts in the vicinity of the fatigue crack focus on the sample surface. The fatigue crack area can be characterised by the river patterns and striations. The dimple structure is characteristic of the unstable crack propagation zone that leads to the rupture of the samples. Between both stable and unstable crack propagation, the transient zone can be found, where both types of crack propagation may be found: dimples and striations together. Saturated strain amplitudes are greater than 0.002, which indicates microplastic deformation of the samples. The acoustic emission signal is registered in the quasistatic compression test points to the twinning activity induced by a small stress. Plastic deformation in the compressive half cycle starts with the formation of extension twins $\left\{10\bar{1}2\right\}\;\langle 10\bar{1}1\rangle$. In the samples with fibre basal texture, the basal (0001) planes are oriented parallel to the extrusion direction, i.e., the Schmid factor of these planes is small, and the dislocation slip is less likely than twinning. As was mentioned previously, the critical resolved stress for nucleation and the formation of extension twins is higher in tension compared with those measured in compression [42]. Twins are formed in the compression half cycle are erased in the next half cycle. Li et al. pointed out that twinning and detwinning play an important role during the fatigue deformation process [55]. With the increasing number of cycles, the internal localised stresses increased, thus increasing the number of sites where twins are nucleated and also the number of residual twins. They may pile up and form microcracks. Cracking of the boundaries of the twins is caused by the incompatibility of the stress between the twins and the basal dislocations. Yin et al. [56] studied the morphology of fracture surfaces in AZ31 samples, subjected to low cycle fatigue. They estimated that the stable propagation period can be characterised by the twinning–detwinning mechanism, while the final failure is realised mainly by the slip of dislocations. Thus, the cooperation of twinning and dislocation slips is the main mechanism leading to the fatigue fracture.

## 5. Conclusions

Rotary-swaged AZ31 alloy was submitted to symmetric low-frequency cyclic push–pull loading. The following conclusions may be drawn. Rotary swaging and subsequent thermal treatment substantially refined and homogenised the microstructure. Tensile characteristics were substantially improved compared with those of the extruded alloy. The fatigue properties were improved—the endurance limit based on 10^7^ cycles was approximately 120 MPa. This value is much higher compared to the AZ31 alloys prepared with other methods of severe plastic deformation. The analysis of the fracture surfaces showed three zones: zone of the fatigue fracture, zone of the final fracture, and a transient zone between them. The transcrystalline fatigue fracture was found broken in all samples due to the cyclic loading. 

## Figures and Tables

**Table 1:** Deformation characteristics of extruded and swaged samples.

Sample	TYS (MPa)	UTS (MPa)	CYS (MPa)	CPS (MPa)	Tensile Strain	Compr. Strain
extruded	102	341	98	374	0.20	0.09
swaged	154	329	186	428	0.17	0.13
swaged + aged	244	343	209	567	0.13	0.11

## Data Availability

Not applicable.
